# Differences in Game Meat Consumer Behaviour in a Game Meat-Producing Region: The Case of Andalusia

**DOI:** 10.3390/foods14234021

**Published:** 2025-11-24

**Authors:** Pedro Pablo Pérez Hernández, José Manuel Martín Lozano, Miguel Romero Velasco, Pilar Algaba Cenizo

**Affiliations:** 1 Department of Economics, Loyola University of Andalucia, 14004 Córdoba, Spain; 2 Department of Humanities and Philosophy, Loyola University of Andalucia, 14004 Córdoba, Spain; jmmartin@uloyola.es; 3 Department of Law, Loyola University of Andalucia, 14004 Córdoba, Spain; mromero@uloyola.es; 4 Data Unit, Loyola University of Andalucia, 14004 Córdoba, Spain; palgaba@uloyola.es

**Keywords:** Andalusian, consumer behavior, game meat

## Abstract

The product of game or wild meat has been analyzed from a nutritional, ecological, and economic perspective in numerous studies. Consumers have various opinions regarding this source of meat due to a number of reasons. In our research, based on a survey of more than one thousand consumers, we reveal the characteristics of these consumers in a region producing a significant quantity of game meat, Andalucia. However, despite a significant production of this meat in Andalucia, consumption appears surprisingly low, as approximately 90% of this meat is exported. Consumer attitudes to this source of meat are both varied and complex, and a full understanding of the reasons for this remains lacking. In summary, this study attempts to reveal the profile of the Andalusian consumer and actions that should be taken, both in public administration and the production sector, to increase the consumption of this food source.

## 1. Introduction

### 1.1. Context and Importance

Hunting has been and remains an inherent human activity. However, its level of acceptance has varied and been the subject of much discussion around the world [1,2]. Nevertheless, at both a global and European level, significant quantities of game are produced, traded, and consumed. Although this activity is extensively monitored, the focus of this monitoring tends to be based on environmental and nutritional factors rather than economic importance [3].

In Spain, hunting has both supporters and detractors and has been the subject of numerous studies [4,5]. In Andalucia alone, hunting covers an area of 8,726,800 hectares, equivalent to 17.3% of Spanish territory, which is comparable in size and complexity to several other European countries. In terms of hunting activity in Andalucía, seven million hectares of land is enclosed, accounting for 7521 hunting zones, which corresponds to 20% of Spain’s total surface area and 80% of the region’s territory. More than five million (149,926 big and 5,026,287 small) game animals were hunted during the 2022 season in Andalucia, and one hundred and sixty-two thousand hunting licenses were granted in 2024, of which 6% were granted to nonresident hunters in the region [6]. Both hunting and sanitary regulations regarding game meat in Andalucía [7,8,9] are strict and correspond to many European countries [10,11].

All data regarding this activity indicates its economic importance, particularly in rural areas with scarce resources and a reducing population and illustrates how hunting is vital in maintaining the economy and population in these areas [12,13]. Another advantage of this activity is helping balance the existing ecosystem in areas where hunting is carried out [14].

Various studies have focused on consumer attitudes toward game meat, particularly in Eastern Europe [15,16,17] and Mediterranean countries [18].

The obtained results reflect game meat consumer characteristics, demand for this produce, and consumer attitudes. Equally, in Spain, various studies have been carried out during different periods focusing on the consumption of this product in the country [19]. However, to date, in Andalucia, no specific study has been carried out on consumer behavior toward game meat and why consumption is so low.

### 1.2. Objectives of the Study

The main objective of our study is to analyze the behavior of Andalusian consumers toward game meat. The concept of the consumer has been clearly defined by certain authors [20], who have claimed that a consumer is a person who identifies their needs or desires, and once these needs and desires have been identified, acquires a related product or service. Undoubtedly, information about the intention of a consumer in relation to a product or service has been studied in numerous investigations and by many authors from the 1950s to the present, especially with respect to investigating the reasons that lead them to demand or not demand a certain good or service. In fact, knowing the skills and behavior of consumers is necessary for companies so that they can guide their production, determine ways to make their product known, and/or provide sufficient information to the consumer to ensure that the consumer has total clarity about the characteristics of an offered product. More specifically, for products that are related to human nutrition, such as meat, companies must consider the reasons for the corresponding demand, which in addition to price, can include the nutritional value, ecological value, environmentally friendliness value, and sustainability of a product [21]. In this sense, a good study on the consumption of meat (not specifically game) highlights the characteristics of a product that causes a consumer to consume a type of meat and/or select meat based on its production process [22]. Although some studies have indicated that, in general, meat consumption has decreased in recent years, others note the opposite, thus making it easy to categorize by type of consumer and their preferences [23]. On the other hand, some studies have indicated that natural and ecologically friendly meat consumption is more sustainable and is beginning to be accepted by consumers to a greater extent [24,25].

The goal of our research is to determine the behavior of the consumers in the autonomous community of Andalusia with respect to game meat. Undoubtedly, compared with other regions of Spain or Europe, the Andalusian region produces a large amount of game meat, which is appealing to meat companies that specialize in this type of wild meat. However, it is estimated that approximately 90% of the game meat obtained in Andalusia is exported outside of Spain [26]. The low demand exhibited by Andalusian and/or Spanish consumers is reflected in the statistics of food consumption in the country. Studies show that game meat does not appear individually but is rather included in the heading “other meats”, which accounts for less than one kilogram/person of meat consumption per year come to more than thirty kilograms/person for farm meat [27]. Given this situation, to identify the reasons behind this level of consumption of this product in Andalusia, we propose the following three specific objectives:

(a) To know and understand the preferences and opinions of Andalusian consumers regarding wild game meat and its incorporation into their diet;

(b) Identify the attitudes of consumers in this region toward the consumption of game meat/dishes;

(c) Identify, describe, and compare the different types of Andalusian consumers with regard to game meat, and what actions could modify their behavior to increase consumption.

This analysis will be very useful for efforts to implement relevant measures or plan new marketing strategies that can increase the consumption of this type of meat in Andalusia, as the attributes of this meat positively affect human health according to scientific studies [28].

## 2. Materials and Methods

To achieve the proposed objectives, a representative survey of the population in the region was conducted (the area in question consisted of a total of 8,631,862 inhabitants in 2024 [29]), in which context relevant characteristics, both of the total population and its distribution throughout the territory, were considered. The method used to obtain the information was a computer-assisted telephone interview (CATI), which has been used in similar studies on the behavior of game meat consumers [30,31]. The survey was conducted in the third quarter of 2023 and administered through software that presented the questionnaire to the respondents.

Once the survey was completed, it was validated, and all the information obtained was analyzed. Before the survey was conducted, the questionnaire was tested by reference to a sample of twenty people, and any errors identified by the interviewees were corrected.

Thus, as Table 1 shows, the survey involved a total of 1066 consumers who contributed approximately eighteen thousand responses. The information collected in the table shows the robustness of the survey from a statistical perspective, which conveys the robustness of the results, and in turn, allows valid conclusions to be drawn with the goal of achieving the proposed objectives. The respondents were completely anonymous and chosen at random, and at no time did the participants provide contact information (including Internet protocol (IP) addresses), in accordance with the General Data Protection Regulation of the European Parliament [32].

The questionnaire used in this research consists of a total of 15 questions, alongside a final section that features five questions regarding respondents’ general information (see Supplementary Materials). To develop the questionnaire, other previous studies conducted both in Spain and elsewhere were considered [4,19,33] and adapted to identify the characteristics of consumers in our region as well as their attitudes toward game meat.

First, an analysis of the data provided through different contingency tables was conducted, which allowed us to determine the distribution of the responses of the respondents regarding their consumption or nonconsumption of game meat.

Once the information concerning each of the questions had been obtained, the variables that defined the characteristics of the individuals who identified themselves as consumers or nonconsumers of game meat were compared. For this step, the statistical analysis of the results was performed using R software [34]. The chi-square test was used in this study to assess the influence of factors describing the population on the examined features. The significance of differences between the values was determined at a significance level of *p* ≤ 0.05.

The Mann–Whitney U test was used to assess the influence of consuming/not consuming wild game on sociodemographic factors [16,35]. The null hypothesis was the lack of significant differences between the distributions of factors in both groups; the alternative hypothesis was the significant differentiation of factors caused by eating wild game. The test was performed at *p* ≤ 0.05.

Once the results obtained were analyzed, we created groups of respondents with a similar attitude toward game consumption. The respondents were divided into two groups: people who consumed wild game and those who did not. This approach allowed us to differentiate between the characteristics of each group more clearly and examine the similarities and differences between the two groups.

## 3. Results

The main characteristics of the respondents are presented in Table 2. The interviewees (1066 people) were distributed between those who reported that they were consumers of game meat (280, 26.3%) and those who reported that they were not consumers of this type of meat (786, 73.7%) in the past year; in addition, some of the participants had not tried this product, as noted later. With respect to the age of the interviewees, adults older than 20 were considered, and the 40–64 age group accounted for the greatest number of respondents (48%), although those in the first interval (i.e., the 20–39 age group) declared that they consumed a greater amount of game meat than the other participants. In other studies, the age of the consumers was similar than to that considered in this study [36]. On the other hand, among the total sample, 45.4% of participants were residents of the capital of each of the Andalusian provinces, whereas 54.6% were residents of the towns or villages located in each of the eight provinces in the region.

In terms of gender, more women (51.2%) than men (48.8%) were interviewed, although the latter reported that they consumed wild meat to a greater extent than the female participants [31,37]. Similarly, most respondents (61%) were at the average socioeconomic level, 31% of respondents were at the medium–low level, and 9% were at the high level 9%, and those in the high socioeconomic category accounted for the proportionally highest consumption of wild meat (36.7%). Finally, in terms of education level of the respondents, 68% of those who responded to the survey completed high school or vocational training (392, 37% of the total) or higher university studies (330, 31% of the total). This information indicates that the distribution of the sample surveyed is consistent with the characteristics of the Andalusian population.

### Reasons for Consuming or Not Consuming Game Meat

The responses provided by Andalusian consumers concerning their level of consumption showed that 73.7% of those surveyed had not consumed game meat during the past year, while 26.3% had done so at some point. However, respondents who indicated that they had not tried game meat in the past year were asked whether they had ever tried this type of meat; only 31.7% answered that they had never consumed it. In fact, 68.3% of the respondents had tried this product at some time in their life, thus indicating that there is an opportunity to increase the consumption of this type of meat in the future.

On the other hand, only 6.1% of those who responded that they consumed game meat indicated that they were weekly consumers of this product; that is, they usually consumed this type of meat once per week, while 16% and 9% indicated that they usually consumed it 4 and 5 times per year, respectively. These data indicate the minimal consumption occurs in the Andalusian region, despite its status as a large producer of game meat.

In terms of the reasons why game meat is not consumed, each group was asked to report their opinions separately, and the results are presented in Table 3. Respondents who identified as nonconsumers indicated that they were not in the habit of eating this type of meat as the main reason for their lack of consumption (16, 31% of the total answers given), followed by the difficulty of finding this type of meat (14.76%) [38], opposition to hunting (12.07%), and to a lesser extent, issues related to flavor (9.38%). Respondents noted that they did not consume this type of meat as they were opposed to hunting [39], and this reason was the most powerful explanations of their lack of consumption of this type of meat; furthermore, this factor was associated with ignorance of the benefits of hunting with respect to the maintenance of the ecosystems where game animals live [40,41].

On the other hand, respondents who did consume this type of meat regularly indicated that they did not do so because of a lack of information (19.91%), and second, because of the difficulty of finding this type of meat in the market (15.58%). The latter reason is striking because it is exactly the same as the explanation provided by nonconsumers. A smaller number of respondents indicated that they were not in the habit of eating wild game or that they lacked knowledge about this type of meat, so they had never or rarely tried it (10.74%).

The remaining reasons provided by both groups (consumers and nonconsumers) about why they believe this type of meat is not consumed were similar, albeit with different percentages in each group.

In contrast, when those who were consumers of this type of meat were asked about their reasons for its consumption, the main reason they indicated they consumed it was for its flavor (28.81% of the total answers given) [42], followed by habits (19.91%), because they preferred this meat (12.65%), because of family tradition (12.65%), and because they considered it to be more natural (11.94%) or healthier (8.90%) [43]. At lower percentages, they indicated that they consumed this type of meat because they were hunters [44], relatives of hunters or knew hunters (3.98%). In terms of hunters that consume game meat, it is interesting to identify how and why they consume this type of meat, behavior that differs from that of other consumers and with which there is a strong identification [45].

On the other hand, the majority of game meat consumers (49.2%/280) reported that they prepared this food at home or at the home of friends or relatives (10.4%/280). It is therefore significant that consumers treat game meat like any other type of farmed meat. Finally, 31.7% (280) of consumers consumed this product in restaurants that specialize in this type of food.

Some questions were also included in the questionnaire to enable the respondents to provide their opinions on different aspects ranging from the European name (it is called wild meat instead of game meat) [46] to the generation of economic activity by hunting to nutritional and health aspects with respect to game meat. The results are presented in Table 4. Responses pertaining to the degree of approval of changing the name of game meat to wild meat (Table 4.A) indicate that 14% were against such a change, while 40% viewed it as a good or very good idea (accounting for more than 50% among those who identified as consumers and for 36% for nonconsumers).

Regarding the potential of game meat to serve as a more ecologically and environmentally friendly product [47] than other farm meats (pork, veal, and poultry) (Table 4.B), 54% of the respondents agreed with this statement (66% of game meat consumers versus 49% of nonconsumers), whereas 17% disagreed (13% of consumers versus 19% of nonconsumers). Thus, when certain information regarding the ecological value of this type of meat production is provided [48], the vision and attitude of the consumer become more favorable, irrespective of whether the respondents in question are consumers of game meat [49]. Similarly, the benefit of hunting in terms of maintaining the ecological balance of areas where this activity is practiced is a key element to people’s ability to understand and accept hunting, which undoubtedly helps to modify the attitudes of consumers toward wild meat [50,51].

Other relevant information was obtained from the answers to the questions, which is included in Table 5. The first question (Table 5.A) included elements that explained positive aspects of the health of game meat.

Thus, with respect to this first question, the respondents indicated that, according to nutrition experts, wild meat contains more fiber and less fat than other meats that are commonly consumed [52,53,54,55,56,57]. It is also a more natural and ecologically friendly meat since the animals are not confined and are not fed or fed small quantities of feed or fattening products [58]. Approximately 73% of the respondents considered this information very interesting (82% of consumers compared to 69% of nonconsumers), whereas slightly less than 5% considered it minimally or not at all interesting.

The second question presented in Table 5.B asked the interviewees to comment on the economic aspects that the game meat sector generates in our country: approximately 54,000 direct annual jobs generated by hunting and jobs involving the sector directly or indirectly affect more than 5 million people in the country, especially in rural areas [59]. In addition, game meat is highly valued in Europe, to which approximately 90% of the annual production of such meat is exported [4,19]. This information caused 73% of the respondents to note that they found the information interesting or very interesting (71% of nonconsumers and 79% of consumers); however, 5.5% answered that they did not find it interesting, and 4.4% did not know what to say.

The respondents were subsequently asked whether an accreditation or certification of the quality of game meat [60] that identified its traceability, provenance, and quality assurance would lead them to increase their consumption or try this product (Table 6). Their responses to this question maintained the same proportion as their answers to the previous questions. Thus, 29.3% of the respondents indicated that they would not consume this product under any circumstances, although the remaining respondents indicated that they would begin to consume it, maintain their current level of consumption, or increase their consumption.

Finally, all respondents were asked how they would prefer to consume game meat (Table 7), regardless of whether they had consumed this product at some point or not. The answers were that 21.5% indicated that they would not consume it under any circumstances, but 79.5% indicated that they would consume it in different ways. Thus, the option of cooking it in their own home was the most common response (30%), followed by consuming it in restaurants (23.6%), or indifference toward consuming it at home or in restaurants (21.6%). Thus, the results of this study are consistent with those of other studies [61] by indicating that if certification was implemented by political leaders and competent authorities, it could serve as a valid tool for promoting the purchase of wild game meat.

To further explore the significance of these differences, the Mann–Whitney U test was applied to ordinal variables. The results are summarized in Table 8. While age did not show a statistically significant difference between consumers and nonconsumers (*p* = 0.1083), the socioeconomic level and educational level did. Consumers tended to have a higher socioeconomic status (*p* = 0.0001) and a higher level of education, such as a baccalaureate degree (*p* = 0.0378), compared to nonconsumers, who were more likely to have no formal studies.

Thus, with the aim of exploring the significance of the differences observed in the information obtained in this research, in Table 8, we considered the different variables included in the survey and their levels of significance according to different statistical tests that were performed in accordance with the type of variable in question.

The reasons for consumption or the nonconsumption of wild game meat were found to be significantly associated with certain sociodemographic factors (*p* < 0.05). A statistically significant relationship was observed between consumption and province of residence (*p* = 0.02954), suggesting that regional traditions or availability may influence dietary choices. Likewise, residence type (urban vs. rural) showed a significant association (*p* = 0.01373), indicating that individuals living in rural areas may be more inclined to consume wild game, possibly due to cultural or environmental factors [62].

Moreover, the combined variable socioeconomic level and residence revealed a strong relationship with consumption patterns (*p* = 0.005065), suggesting that both financial status and living environment jointly affect attitudes toward wild game meat. In contrast, gender did not show a statistically significant influence on consumption (*p* = 0.5857), indicating that men and women in the sample had similar behaviors regarding wild game meat.

These findings could imply that different socioeconomic levels and types of residence are associated with different game meat consumption patterns. To visualize this situation more effectively, Table 9 presents the different economic levels and places of residence of the respondents.

The data revealed that those who had higher incomes and who lived in the towns of the Andalusian provinces (where there is greater knowledge and proximity to rural areas) had a higher level of consumption (42.30% of consumers of the total), whereas those whose income was lower and whose place of residence was the capital of the province consumed less game meat (17.86% of the total number of respondents).

One analysis that we considered very important involved comparing the reasons given by both groups (consumers and nonconsumers) concerning why the consumption of game meat was so low in Andalusia (as shown in Table 3). As indicated, each group was asked this question separately, and the answers were open and multiple. Thus, those who reported themselves as consumers provided a total of 447 reasons, which represented a total of 17 different answers and accounted for 98.9% of the answers given. On the other hand, nonconsumers reported a total of 1226 reasons that represented 14 different responses, accounting for 96.2% of the total responses. Nonetheless, the number of different answers given by the respondents (17 and 14 from consumers and nonconsumers, respectively) was considered to be excessively dispersed in terms of the reasons offered by each group. Thus, the responses were recoded, leaving a total of four groups of responses that were common to each group (Table 10).

Once the respondents’ reasons for not consuming game meat were reduced to four, it was easy to balance the number of responses given by each group, since the difference was more than remarkable. Accordingly, the consumer data were adjusted so that they had proportionally the same number of responses as that given by the nonconsumers, resulting in a new column (consumers adjusted). In this way, possible bias was prevented when the chi-square test was performed. The application of this test resulted in a *p* value ≤ 0.001, which explains the significant differences between the two groups; therefore, the type of consumer influences the responses given, and the differences observed were not the product of chance.

## 4. Discussion

The survey data provided extensive information on various aspects to consider regarding consumer characteristics and their behavior toward this product. It shows that the youngest group of consumers (aged 20 to 39) and males consumed more game meat, largely due to this group’s involvement in hunting and trying a new flavor of a little-known meat. However, gender did not show a statistically significant influence on game meat consumption when considering the total number of respondents.

It is also striking that among the reasons given by consumers, location (i.e., in villages, where hunting is a common activity and consumption levels tend to be higher) or having a family member or acquaintance who is a hunter had such insignificant effects [44]. This behavior differs from that observed in other countries, where game meat consumption is higher in these areas or is known through people who participate in hunting and obtain this type of meat [63].

On the other hand, based on the reasons given by nonconsumers, some significant factors can be identified. Firstly, those who do not consume this type of meat gave reasons that could be used to help them change their perceptions and modify their meat consumption habits. For example, they are not accustomed to eating game meat, they find it difficult to find this type of meat, they do not like its taste (an aspect that is highlighted not only in our region but in many other countries) [64], mistrust regarding the origin and quality of the meat, and lack of knowledge about how to cook it are factors that could be used in a marketing campaign (by private companies and/or public administration) to influence the perceptions of nonconsumers, thus encouraging them to change their attitude toward game meat.

It is noteworthy that 50% of consumers of game meat stated that they encountered no difficulty in locating this product in the market, thereby indicating that regular consumers have ready access to this particular type of meat. In this regard, it was determined that on a scale of 1 to 5 (1 being no difficulty, 5 being maximum difficulty), the average value obtained was 2.289, with a standard deviation of 1.49. These findings suggest that 75% of consumers of game meat may be able to locate this type of meat with minimal effort. Consequently, a reasonable conclusion that can be deduced from the responses of 20% of the Andalusian population surveyed is that the product is readily available within the commercial sphere. It is acknowledged that for the general population, discerning this phenomenon can be challenging. This is primarily due to the fact that the majority of consumers purchase goods in supermarkets, where the product is not available, thereby hindering the process of discovery.

In the context of the survey, respondents who had abstained from consuming game meat during the previous twelve-month period were posed a query regarding their potential previous consumption of such meat. The data collected from the survey revealed that 68% of the subjects surveyed reported having consumed the substance at least once, suggesting that they were already aware of its existence prior to the survey. These data are of great significance within the regional context, insofar as they indicate that 50% of the subjects surveyed reported neither regular consumption of game meat, nor its consumption in the past. Within the context of the investigation, it was observed that in conjunction with subjects demonstrating a high frequency of consumption of game meat, 78% of the surveyed population had, at some point, consumed this type of foodstuff. Nevertheless, it should be noted that not all subjects were habitual consumers. In contrast, 32% of respondents who did not consume game meat had never previously sampled it.

The survey results show that 47% of nonconsumers said that a lack of culture or tradition, the taste of the meat, or how it is cooked were the reasons why they did not consume game meat, while 68% of consumers said that a lack of information, advertising, and promotion of game meat were the reasons why this type of meat is not consumed. Based on the information provided by the respondents, we can determine that approximately 71% of nonconsumers would be potential consumers of game meat if they had more information about the health benefits of this meat and could access it more easily, a situation that does not currently exist in our region. This suggests that game meat in our autonomous community has the potential to be introduced as a dietary staple, even as a replacement for other types of meat.

Finally, the information provided by the interviewees shows that 17% of those who declared that they had never consumed it did so because they opposed the hunting activity, which is undoubtedly a difficult attitude to change for this group of consumers. In our opinion, this group is very small in relation to the total number of potential consumers of game meat.

### Limitations

The primary strength of our study lies in the substantial and representative sample of the Andalusian adult population (*n* = 1066), which is commensurate with the region’s total population. The selection process incorporated a comprehensive evaluation of all the factors that exert influence on the consumer. It is accurate to state that the survey did not include questions regarding the type of game meat consumed. This limitation restricts the ability to differentiate between different species, which would have enhanced the precision of the results. In addition, a greater division of the age groups would have resulted in a more detailed breakdown of the population under study.

It is also important to highlight that our results are specific to the cultural context of Andalusia and should be interpreted with caution in relation to other regions or countries. Notwithstanding the limitations, the results obtained demonstrate a high degree of accuracy in relation to the consumption habits of Andalusian consumers with regard to wild game meat from Andalusia, which has not been the subject of study hitherto, particularly in a region that is predominantly engaged in the production of wild game meat.

## 5. Conclusions

The autonomous community of Andalusia, located in southern Spain, is renowned for its enthusiasm for hunting. This article presents a comprehensive analysis of the behavior of consumers and nonconsumers of game meat, as well as their respective consumption patterns for this product. The initial finding from this analysis is that the number of consumers is excessively low, which is the reason why game meat companies must export approximately ninety per cent of their production mainly to Central European countries.

Secondly, the information gathered highlights consumers’ lack of knowledge about how wild meat is obtained, its health benefits compared to other farm-raised meats, and the many advantages that hunting activities bring to the economy of the producing areas. This aspect is even more striking in a region that is a major producer of game meat.

This study has thus revealed the measures that could be implemented in order to increase the consumption of game meat among the population. It is imperative that public administration, on the one hand, and the various actors involved in the production of wild meat (companies, hunters, hunting ground owners, among others), on the other hand, implement various measures that, in the opinion of consumers, would improve their consumption of game meat. Such measures could include quality certification, greater availability of wild meat in supermarkets, promotion of the benefits of the product, and the contribution of hunting to the economic improvement and environmental balance of the territories where game meat is produced.

## Figures and Tables

**Table 1:** Survey characteristics.

Sample description	General population over 20 years of age
Scope of study	Community of Andalusia
Sample size	1066 interviews
Survey fieldwork procedure	Computer-assisted telephone interview (CATI) by sampling (INE, 2022)
Sampling unit	Representativeness by gender, age and province
Sampling mode	Simple random sampling
Statistical reliability	97% confidence level and sampling error 3.3%
Study variables identified	17,896 responses analyzed
Dates of survey work	1 August 2023–28 August 2023

Source: Own elaboration.

**Table 2:** Sample and characteristics of the respondents.

Population Features	Game Meat Consumer (*n*, %)	Not a Game Meat Consumer (*n*, %)
Sample	280 (26.27%)	786 (73.73%)
*Age*		
20–39 years old	93 (29.43%)	223 (70.57%)
40–64 years old	131 (25.69%)	379 (74.31%)
>64 years old	56 (23.33%)	184 (76.67%)
*Locality of residence*		
Provincial capital	109 (22.52%)	375 (77.48%)
Village	171 (29.38%)	411 (70.62%)
*Gender*		
Women	139 (25.46%)	407 (74.54%)
Men	141 (27.12%)	379 (72.88%)
*Socioeconomic level*		
High or medium–high	36 (36.73%)	62 (63.27%)
Medium	176 (27.08%)	474 (72.92%)
Medium–low or low	68 (21.38%)	250 (78.62%)
*Education level*		
No studies	2 (8.70%)	21 (91.30%)
Primary School	31 (29.81%)	73 (70.19%)
Secondary School	56 (25.81%)	161 (74.19%)
Baccalaureate or VET	112 (28.57%)	280 (71.43%)
College Degree	41 (26.45%)	114 (73.55%)
Bachelor’s Degree	35 (21.08%)	131 (78.92%)
PhD	3 (33.33%)	6 (66.67%)

Source: Own elaboration.

**Table 3:** Reasons for consuming and not consuming game meat.

Nonconsumers		Consumers	
Reason	%	Reason	%
Lack of habit	16.31	Lack of information in general	19.91
Difficulty finding it in supermarkets/restaurants	14.76	Difficulty finding it in supermarkets/restaurants	15.88
Mainly against hunting	12.07	Lack of custom	10.74
Because of the taste	9.38	Consumers are unknown, have never or rarely tried it	10.74
Not included in regular family menu	7.26	Lack of promotion, advertising, and publicity	9.84
No culture of consuming this type of meat	6.61	Price	8.28
Does not eat meat in general	5.46	No culture of consuming this type of meat	6.49
Tried it and did not like it	5.30	Prejudices about the world of hunting	6.26
Price	5.30	Consumed by a very small minority	2.68
Tough meat	4.32	Not fashionable	2.46
Distrust in quality and origin	3.43	Because of the taste/dislike	1.34
Family does not like it	2.94	Only consumed on special occasions	1.34
Rarely consumed by certain groups	1.79	Not regularly included in family menu	1.12
Lack of awareness of how to cook it	1.22	Mistrust of quality and origin	0.89
Other reasons	3.83	Does not consume meat in general	0.45
		Other types of meat preferred	0.45
		Other reasons	1.12

Source: Own elaboration.

**Table 4:** Level of agreement with the wild (4.A), organic and environmentally friendly (4.B) meat designation.

A				B			
Items	Nonconsumers	Consumers	Total	Items	Nonconsumers	Consumers	Total
Looks very bad	43 (5.47%)	9 (3.21%)	52 (4.88%)	Strongly disagree	66 (8.4%)	13 (4.64%)	79 (7.41%)
Thinks it is wrong	76 (9.67%)	18 (6.43%)	94 (8.82%)	Disagree	81 (10.31%)	23 (8.21%)	104 (9.76%)
Does not care	380 (48.35%)	110 (39.29%)	490 (45.97%)	Does not care	162 (20.61%)	35 (12.5%)	197 (18.48%)
Thinks it is fine	266 (33.84%)	123 (43.93%)	389 (36.49%)	Agree	264 (33.59%)	129 (46.07%)	393 (36.87%)
Thinks it is very good	20 (2.54%)	20 (7.14%)	40 (3.75%)	Strongly agree	124 (15.78%)	63 (22.5%)	187 (17.54%)
Does not know/no answer	1 (0.13%)	–	1 (0.09%)	Does not know/no answer	89 (11.32%)	17 (6.07%)	106 (9.94%)

Source: Own elaboration.

**Table 5:** Opinion on health benefits (A) and benefits for employment and the economy (B).

	A				B	
Items	Nonconsumers	Consumers	Total	Nonconsumers	Consumers	Total
Very interesting	154 (19.59%)	73 (26.07%)	227 (21.29%)	162 (20.61%)	83 (29.64%)	245 (22.98%)
Interesting	391 (49.75%)	156 (55.71%)	547 (51.31%)	395 (50.25%)	137 (48.93%)	532 (49.91%)
Does not care	203 (25.83%)	40 (14.29%)	243 (22.80%)	140 (17.81%)	43 (15.36%)	183 (17.17%)
Not very interesting	24 (3.05%)	11 (3.93%)	35 (3.28%)	31 (3.94%)	10 (3.57%)	41 (3.85%)
Not interesting at all	14 (1.78%)	–	14 (1.31%)	18 (2.29%)	–	18 (1.69%)
Does not know/no answer	–	–	–	40 (5.09%)	7 (2.5%)	47 (4.41%)

Source: Own elaboration.

**Table 6:** Accreditation or certification of the quality of game meat and level of consumption.

Items	Nonconsumers	Consumers	Total
I would continue not to consume it	305 (38.80%)	7 (2.50%)	312 (29.27%)
I may start consuming it	212 (26.97%)	11 (3.93%)	223 (20.92%)
I would consume it as I do now	88 (11.20%)	131 (46.79%)	219 (20.54%)
I would probably consume it slightly more	120 (15.27%)	67 (23.93%)	187 (17.54%)
I definitely would increase my current consumption	61 (7.76%)	64 (22.86%)	125 (11.73%)

Source: Own elaboration.

**Table 7:** Places to eat more game meat than you do today.

Items	Nonconsumers	Consumers	Total
I would not eat it at all	224 (28.50%)	5 (1.79%)	229 (21.48%)
Mainly prefer to eat it at home cooking it	189 (24.05%)	131 (46.79%)	320 (30.02%)
Preferably in traditional restaurants/taverns	189 (24.05%)	63 (22.50%)	252 (23.64%)
At home or restaurants without distinction	143 (18.19%)	87 (31.07%)	230 (21.58%)
At the house of friends or relatives who know how to cook it	86 (10.94%)	33 (11.79%)	119 (11.16%)
In other places	6 (0.76%)	2 (0.71%)	8 (0.75%)

Source: Own elaboration.

**Table 8:** Associations of the categorical variables.

Population Feature	U Statistic	*p*-Value	Median (No Consumption)	Median (Consumption)
Age [years]	35,608.00	0.1083	20–39	>64
Socioeconomic Level	121.382	0.0001	Medium–Low/Low	High/Medium–High
Education Level	1448.50	0.0378	No studies	Baccalaureate

Source: Own elaboration.

**Table 9:** Consumption patterns of game meat.

Socioeconomic Level	Residence	Nonconsumers	Consumers	% Consumers/Total
High	Capital city	32	14	30.43%
High	Province	30	22	42.30%
Medium	Capital city	228	70	23.48%
Medium	Province	246	106	30.11%
Low	Capital city	115	25	17.86%
Low	Province	135	43	24.15%

Source: Own elaboration.

**Table 10:** Recoding of reasons for not consuming game meat.

No.	Clustered Response	Consumers	Consumers Adjusted	Nonconsumers
1	Absence of information, advertising and dissemination	306	839	293
2	Absence of culture, customs, taste, know how to cook it	105	288	575
3	Distrust of quality and preference of other meats	6	17	143
4	Opposition to hunting and preference not to eat meat	30	82	215
	Total	447	1226	1226

Source: Own elaboration.

## Data Availability

The original contributions presented in this study are included in the article. Further inquiries can be directed to the corresponding author.

## Supplementary Materials

The following are available online at https://www.mdpi.com/article/10.3390/foods14234021/s1.
