# Supplementary material for: Differences in Game Meat Consumer Behaviour in a Game Meat-Producing Region: The Case of Andalusia
Source: Foods. 2025 Nov 24;14(23):4021. doi: 10.3390/foods14234021 (PMC12692111; doi:10.3390/foods14234021)
Supplement: Supplementary file 1 [file foods-14-04021-s001.zip › foods-3951557-supplementary.pdf]

foods

MDPI

Supplementary Materials

Good morning/afternoon. We are conducting a study to learn about eating habits regarding game meat. This research study aims to analyze what consumers, regardless of whether they eat it or not, know about the properties of game meat. THANK YOU FOR YOUR TIME. It will be very brief.

(1) In your diet or in your household, how would you rate your consumption of game meat in the last year?

1. None☐ 2. Very sporadically☐ 3. Sporadically☐ 4. Regularly☐ 5. Very regularly☐

(Answer 1.- go to Q7.)

(2) Approximately how many times have you or your family members consumed game meat in the last year?

(multiple answers allowed, max. 1)

☐At least once a year
☐At least twice a year
☐At least once or twice every three months
☐Consumed at least once a month
☐Between two and three times a month
☐Between once and several times a week
☐At least once a week

(3) Where do you most commonly consume game meat?
(multiple answers allowed, max. 2)

☐At home, cooking it themselves
☐Mainly in traditional restaurants/taverns
☐Both places, without distinction
☐At the homes of friends and/or family members who know how to cook it
☐Other places, please specify:___________

(4) When consuming it, do you have difficulty finding establishments where it is sold and offered for personal consumption or on restaurant menus? Identify this on a scale where 1 means no difficulty and 5 means great difficulty in finding game meat to consume:

1.☐ 2.☐ 3.☐ 4.☐ 5.☐

(5) What would be the main reasons you would highlight for consuming game meat?
(multiple answers allowed, max. 2)

☐You simply like it more than other meats
☐Because of the taste
☐Because of family tradition
☐Because of custom
☐You are a hunter/you know hunters
☐It is commonly consumed in your area/locality
☐It is more natural, it has no additives
☐It is healthier, it has no fiber
☐Other reasons, please specify below:

(6) In your opinion, what would you say are the main reasons why more game meat is not consumed in our country? Please give me at least two or three reasons *(not prompted to identify the one that comes closest)*

☐Lack of information in general
☐Lack of custom
☐Lack of promotion, advertising, and publicity
☐There is no culture of consuming this type of meat
☐Lack of knowledge; consumers have never or rarely tried it
☐Because of the taste; consumers do not like it
☐Difficulty in cooking
☐Not meat to be regularly included in the family menu
☐Only consumed on special occasions
☐Consumption is very limited to a specific audience
☐Prejudices about the world of hunting
☐Difficulty in finding it in supermarkets/restaurants
☐Because of the price

- [ ] Does not consume meat in general
- [ ] Not fashionable
- [ ] Mistrust of the quality and origin of the meat
- [ ] Other types of meat are generally preferred
- [ ] Other reasons, please specify below: (At the end, go to P9.)

(7) Have you ever tried game meat...?

1. Never ☐ 2. No, as far as I know ☐ 3. Sometimes ☐ 4. Yes, I have eaten it ☐

(8) What are the main reasons why you do not eat game meat? Please indicate at least two reasons.

(not prompted to identify the one that best fits)

(9) Did you know that in Europe, game meat is referred to as wild meat, which is the name given to meat that comes from both large and small game and never from farms? Do you agree with this definition?

1. You think it is very wrong ☐ 2. You think it is wrong ☐ 3. You do not care ☐ 4. You think it is right ☐ 5. I think it is very good ☐

(10) According to nutrition experts, game meat contains more fiber and less fat than other commonly consumed meats. It is also a more natural and ecological meat, as animals in the wild do not consume feed or fattening products, or do so in very small quantities. What do you think of this information?

1. Very interesting		2. Interesting		3. I do not care		4. Not very interesting		5. Not interesting at all	

(11) In some sectors, game or wild meat is being referred to as organic and/or environmentally friendly meat, more so than many other meats that are traditionally consumed in homes or restaurants. Do you agree with this statement?

1. Strongly disagree		2. Disagree		3. Do not care		4. Agree		5. Strongly agree		6. Do not know	

(12) Did you know that game meat in our country currently generates approximately 54,000 jobs per year and directly or indirectly involves just over 5 million people, mainly in rural areas? It is a highly prized meat in Europe, where approximately 90% of annual production is exported. What do you think of this information?

1. Very interesting ☐ 2. Interesting ☐ 3. Does not matter ☐ 4. Not very interesting ☐ 5. Not interesting at all ☐ 6. I do not know ☐

(13) If you ever decided to eat game meat or to eat more game meat than you currently do, where would you prefer to do so? (multiple answers allowed, max. 2)

- [ ] I would not eat it under any circumstances
- [ ] I would mainly prefer to cook it at home
- [ ] Preferably in traditional restaurants/taverns
- [ ] In both places without distinction
- [ ] At the homes of friends and/or family who know how to cook it
- [ ] In other places, please specify: ____________________

(14) In your opinion, would accreditation or certification of the quality of game meat that identifies its traceability, origin, and quality guarantee lead you to try it or consume more game or wild meat than you currently do?

1. I would still not consume it ☐ 2. I might start consuming it ☐ 3. I would consume it as I do now ☐ 4. I would probably consume it a little more ☐ 5. I would definitely increase my current consumption ☐

(15) Finally, do you have any suggestions for promoting the consumption of wild game meat in our country?

- [ ] I do not wish to make any suggestions; I am opposed to hunting.
- [ ] I do not wish to make any suggestions.
- [ ] I do wish to make some suggestions. Please indicate: ____________________

(16) Finally, in order to carry out a statistical survey, could you answer the following question?

a. Gender

1. Male ☐ 2. Female ☐

b. Age

1. 20 and 39 ☐ 2. 40 to 64 ☐ 3. 65 or older ☐

c. Socioeconomic status

1. High or Medium–high		2. Medium		3. Medium–low or Low	

d. Level of Education

	1. No certified education
	2. Up to primary education
	3. Up to secondary education
	4. Up to high school or vocational training or equivalent
	5. Up to university diploma or equivalent
	6. Up to university degree or equivalent
	7. Doctorate

e. Place of habitual residence

1. Provincial capital ☐ 2. Provincial town ☐
